# Relationship between aging and control of metabolic syndrome with telomere shortening: a cross-sectional study

**DOI:** 10.1038/s41598-023-44715-1

**Published:** 2023-10-19

**Authors:** Tarachand Devrajani, Shariq Abid, Hina Shaikh, Iram Shaikh, Durshana Bai Devrajani, Sikander Munir Memon, Ali Muhammad Waryah, Ikram Din Ujjan, Binafsha Manzoor Syed

**Affiliations:** 1 Clinical Research Division, Medical Research Center, Liaquat University of Medical and Health Sciences, Jamshoro, Pakistan; 2 Department of Medicine, Liaquat University of Medical and Health Sciences, Jamshoro, Pakistan; 3 Department of Molecular Biology and Genetics, Medical Research Center, Liaquat University of Medical and Health Sciences, Jamshoro, Pakistan; 4 Department of Pathology, Liaquat University of Medical and Health Sciences, Jamshoro, Pakistan

**Keywords:** Molecular biology, Physiology, Biomarkers, Medical research, Pathogenesis

## Abstract

Aging is considered one of the major risk factors for several human disorders. The telomere plays a crucial role in regulating cellular responsiveness to stress and growth stimuli as well as maintaining the integrity of the Deoxyribonucleic Acid (DNA), and aging leads to the progressive decline in the telomere length (TL) due to continuous cell division. The aim of this study was to determine the relationship between TL and advancing age and the impact of metabolic syndrome (MetS) on TL. Firstly, we determined the association of advancing age and TL, by measuring telomere length (T/S ratio) in healthy volunteers (n = 90). The TL was compared between normal population and patients with metabolic syndrome (n = 298). The age matched controlled and uncontrolled MetS patients (n = 149) were also compared for their TL T/S ratio. The TL showed negative correlation with advancing age, whereas the significant change was observed at the cut-offs of 40 and 70 years defining 40 with longer TL and 70 as shorter TL. The longest T/S ratio at 2.46 was measured at the age range of 1 year in healthy volunteers, while elderly population showed considerably shorter TL. The patients older than 60 years with poor or uncontrolled MetS had shorter TL, as compared to the controlled MetS. In conclusion our findings suggest that TL was negatively correlated with advancing age. Uncontrolled metabolic syndrome appeared to have worsening effects on TL. Telomere length appears to have potential to be used a parameter to determine age. However, further large scale studies are recommended to make firm guidelines.

## Introduction

Aging is genetically characterized as the accretion due to modifications occurring over the period of time^1^, and it is also considered as one of the major risk factors for a number of human disorders^2^. Globally, 150,000 individuals die daily and nearly 2/3rd of them die due to age related factors. Aging process however is not constant, but the process gets modified or expedited due to comorbidities such as, cardiovascular and metabolic disorders.

Aging is a genetically controlled phenomenon, and telomeres are essential element to maintain the integrity of the genome and regulating cellular responses to stress and growth factors^3^. In normal cells, under physiological conditions, the telomere length (TL) decreases with each cell division. Given that TL decreases with each cell division, it progressively shortens with advancing age, therefore can be considered a biological marker of aging. This age-related decline in TL has been associated with a number of age-related diseases, including hypertension, diabetes, cancer, Alzheimer's disease, and many others^4^. In addition, there are number of factors that affect TL including oxidative stress, inflammation, and repetitive cell divisions, which correlate TL to chronological aging and age related diseases^5^. The chronological age may differ from biological age due to comorbidities and other influencing factors. During normal cellular activities, free radicals and reactive oxygen species (ROS) are regularly generated. Excessive ROS formation causes oxidative damage and eventually leads to cell abnormalities and cell death.^6^. Higher ROS levels, mitochondrial malfunction, additional double-strand Deoxyribonucleic Acid (DNA) breaks, and shortening of telomeres are indicators of cellular senescence^7^. Furthermore, it was demonstrated that mitochondrial ROS accelerated telomere-dependent senescence. Additionally, literature has shown a correlation between TL and metabolic disorders, suggesting that this could accelerate cellular aging^8,9^. Previously published studies revealed a significant connection between the metabolic syndrome's components and shortened TL^10,11^. However, it is not yet known if TL predicts a wide range of metabolic disorders over a long period, such as lipid profile, hyperglycemia, or hypertension.

Metabolic syndrome (MetS) includes abnormalities in lipid metabolism, hypertension, obesity, diabetes, and hyperinsulinemia^12^. In middle-aged individuals, MetS is closely correlated with higher mortality rates, the development of atherosclerosis, and excessive visceral fat deposition. Additionally, it is connected to limited physical activity and excessive food consumption. Furthermore, a hereditary predisposition to MetS appears to exist as well^12^.

Since there is evidence available suggesting TL as an indicator of biological age but there is limited evidence available showing cut-offs to define age on TL. Therefore, age has not been considered a biological marker in disease correlations and risk prediction due to the lack of robust detection methods. There is also limited literature available to correlate biological age with major comorbidities influencing the aging process such as metabolic syndrome. Therefore, this study was designed to determine correlation of TL with advancing age and the association of TL shortening with metabolic syndrome in both controlled and uncontrolled cases.

## Material and method

This study was conducted at the Clinical Research Division of Medical Research Center, Liaquat University of Medical & Health Sciences, Jamshoro, Pakistan, between January 2018 and December 2022. Ethical approval for the study was obtained from the Ethics Review Committee of the LUMHS Jamshoro (Ref No. LUMHS/REC/645, dated 26/12/2017), and all experiments were performed in accordance with relevant guidelines and regulations.

A total of 388 human subjects were included in this study and written informed consent for inclusion in the study was obtained from all study participants. Selection of the participants was also based on the availability of the blood sample.

The study was performed in three phases: (i) Validation of the protocol for age determination by using TL in healthy volunteers, then (ii) Correlation of TL with metabolic syndrome patients were measured with advancing age and later, (iii) Comparison of age-matched controlled and uncontrolled metabolic syndrome patients was made with healthy volunteers.

For the protocol development, healthy volunteers (Group A) were selected aged between one year to 100 years (n = 90). Age-matched cases in group B and group C with controlled metabolic syndrome (n = 72) and uncontrolled metabolic syndrome (n = 77) respectively were selected. For the control group (Group D) age-matched healthy volunteers were also selected (n = 149). Metabolic syndrome was diagnosed by using criteria provided by the International Diabetes Federation (IDF) Task Force on Epidemiology and Prevention and the American Heart Association/National Heart, Lung, and Blood Institute (AHA/NHLBI). According to the AHA/NHLBI definition, MetS diagnosis requires three out of the following five criteria:

I. Obesity = waist circumference (WC) ≥ 90 cm within men and ≥ 80 cm in women.
II. Hypertriglyceridemia, triglycerides level ≥ 150 mg/dL.
III. Low high-density lipoproteins (HDL) below 40 mg/dL in men and below 50 mg/dL in women.
IV. Hypertension systolic blood pressure (SBP) ≥ 130 mmHg and diastolic blood pressure (DBP) ≥ 80 mmHg.
V. Fasting blood sugar (FBS) ≥ 100 mg/dL.

Based on these criteria, participants were categorized as controlled MetS when their triglycerides, blood pressure, and FBS levels remained within the normal range, with or without medication.

All the patients and healthy volunteers who agreed to consent to be part of the study went through a clinical examination for MetS, and serological investigations for FBS level and fasting lipid profile were performed. The Glycosylated Hemoglobin (HbA1c) levels and random blood glucose (RBS) levels were also measured in blood, and the duration of diabetes and systematic blood pressure were also evaluated.

### Biological sampling

Study participants ' blood samples (3–5 ml) were collected in Ethylene- di-amine-tetra-acetic acid (EDTA) tubes. Samples were then transported to the laboratory and stored at − 20 °C. Firstly, the five reference DNA (adults) with serial dilutions were used to verify the reaction efficiency.

### DNA extraction from blood

DNA was extracted and obtained from collected blood by commercial kit # 51104 (QIAGEN) QIAamp DNA BLOOD Mini Kit) as per manufacturer’s instructions. 20 µl of Protease solution was added with 200 µl into a 1.5 ml micro centrifuge tube. 200 µl Buffer AL were added and mixed thoroughly by vortex. Later, the samples were incubated at 56 °C for 10 min. Briefly centrifugation was performed to remove drops from the lids of a 1.5 ml microcentrifuge tube. 200 µl ethanol (96–100%) was added, mixed thoroughly by vortex, briefly centrifuged the tube to remove drops from the lid, then the mixture was transferred through a pipette in to Mini spin column (in a 2 ml collection tube) and centrifuged at 8000 rpm for 1 min. Collection tubes with flow-through were discarded. Mini spin column was placed in a new 2 ml collection tube, 500 µl Buffer AW2 was added, centrifuged at 14,000 rpm for 3 min, flow through and the collection tubes were discarded.

A new mini spin column was placed in a 1.5 ml micro centrifuge tube then 200 µl Buffer AE or distilled water were added and incubated at room temperature 15–25 °C for 1 min. Centrifugation was done at 8000 rpm for 1 min, to elute the DNA. Each DNA sample's concentration was confirmed using spectrophotometer (Beckman coulter, INC. Du 800, Spectrophotometer, U.S.A. Conventional thermal cycler 2720 (Applied Bio system) was used to optimise temperature before the assay was moved onto quantitative analysis by real-time PCR (Rotor-Gene Q, R0314104, QIAGEN, Germany).

### Quantitative polymerase chain reaction (qPCR)

Real time PCR or Quantitative PCR (qPCR) was performed by using Platinum SYBR Green qPCR super Mix-UDG, (Invitrogen). The reference DNA was loaded in all runs to control the inter-plate variation. All samples were assayed in duplicate wells, and average values of two measurements (Ct value of Telomere and human beta-globin (HBG)) were used for the statistical analysis. Each reaction well contained 30 µl reaction mixture: 15 µl of master mix with 1 µl ROX dye, 2.5 µl of 10uM forward primers, 2.5 µl 10 µM reverse primers, 10 µl DNA (25 ng) template was used. This method was an extension of basic PCR, including relative quantification of target DNA using (cycle threshold) Ct values (Step One software, v2.3).

Temperatures for telomere PCR: first holding stage for 50 °C for 2 min and 95 °C for 2 min, then 40 cycles were set at denaturation 95 °C for 15 s, annealing at 68 °C for 40 s and extension 75 °C for 3 min and second holding for 72 °C for 40 s. For β-globin reference gene PCR: first holding stage for 50 °C for 2 min and 95 °C for 2 min, then 40 cycles were set at denaturation 95 °C for 15 s, annealing at 56 °C for 40 s and extension 75 °C for 3 min and second holding for 72 °C for 40 s.

The telomere length (TL) of blood samples T/S ratio was calculated by using the formula T/S = Ct telomere/Ct single copy gene (reference gene) as described in detail previously^13–15^.

### Reagents and reagents set-up

#### Standards and primers (oligomers)

Primer sequences used for telomere and single-copy gene (Human β globin, HBG) amplification were:

Primer sequences: Tel F, 5′GGTTTTTGAGGGTGAGGGTGAGGGTGAGGGTGAGGGT3′; TelR, 5′TCCCGA-CTATC-CCTATCCCTATCCCTATCCCTATCCCTA3′; HBG F, 5′GCTTCTGACACAAC-TGTGTTCACTAGC 3′; HBG R, 5′CACCAACTTCATCCACGTTCACC 3′7.

All primers were diluted with 20ul of TE Buffer and stored at − 20°C until required. Working stocks of oligomers were made fresh; dilutions were placed at 4 °C for up to 2 weeks.

### Statistical analysis of data

The Statistical Package for Social Sciences (SPSS, IBM, version 22.0) and GraphPad Prism version 8 were used for data analysis. The continuous data, including age in years and TL, were presented as mean and standard deviation (mean ± S.D). Simple frequency and percentage were computed for the sex, and age groups. Stratification concerning the age with TL was done. For analysis, the age groups were first made in decade-wise fashion and TL was correlated and presented as scatter plots. Kolmogorov and Shapiro–Wilk normality tests were performed before analysis of data. Non-parametric Spearman’s correlation coefficient was used to determine the relationships performed in the validation group, healthy volunteers, controlled MetS and Uncontrolled MetS. Finally, Box-plots were developed for three different segments of age (41–50 years, 51–60 years and 61–70 years), and TL, in all groups with healthy individuals, controlled MetS and uncontrolled MetS. Later, Kruskal–Wallis test with subsequent post-hoc Dunn's test was performed to compare the difference between the groups. A *p*-value ≤ 0.05 was considered as significant.

## Results

Telomere length validation group had 90 healthy subjects of aged ranged from 1 to 100 years (n = 90) (Table 1). A summary of the basic parameters of MetS is presented in Table 2 with total subjects of 298 individual aged ranged 40–70 years, which was divided into three groups including healthy volunteers with mean age 54.8 years while controlled and uncontrolled MetS groups had 55.1 and 54.8 years mean age, respectively. The uncontrolled MetS group had higher Triglyceride levels, SBP, DSP and FBS while lower HDL as compared to healthy volunteers and controlled MetS groups (Table 2).

**Table 1:** Telomere length (T/S ratio) in healthy volunteers at different age.

Telomere length (T/S ratio)	Age (years)		
	1–39	40–69	≥ 70
2.46–1.50	57	1	–
1.49–0.62	8	16	–
0.61–00	2	2	4
Total	67	19	4

**Table 2:** Stratified analysis of components of MetS syndrome measured in healthy volunteers, controlled and uncontrolled MetS subjects.

	Healthy volunteers (n = 149)			Controlled MetS (n = 72)			Uncontrolled MetS (n = 77)		
Male/female	74/75			36/36			41/36		
	Mean	Range	SD	Mean	Range	SD	Mean	Range	SD
T/S ratio	0.95	0.32–1.54	0.3	0.93	0.48–1.54	0.3	0.92	0.53–1.54	0.2
Age (years)	54.8	40–70	9.4	55.1	40–70	8.9	54.8	40–70	8.0
WC (cm)	79.83	71–89	4.9	89.80	75–110	7.9	100.27	72–150	13.0
Triglycerides (mg/dL)	132.64	109–150	6.9	162.47	110–270	10.9	209.51	122–378	43.7
HDL (mg/dL)	47.15	30–57	5.8	46.33	30–57	6.5	38.72	15–64	14.8
SBP (mmHg)	117.32	105–125	2.5	121.03	110–140	4.4	142.83	120–180	10.0
DBP (mmHg)	72.38	70–75	10.4	78.21	70–90	36.3	89.42	75–120	60.8
FBS (mg/dL)	89.81	74–99	6.7	106.63	88–130	7.0	171.34	110–300	7.7

WC, waist circumference; HDL, high density lipoproteins; SBP, systolic blood pressure; DBP, diastolic blood pressure; FBS, fasting blood sugar.

### Correlation between telomere length and increasing age

Telomere length was measured in 90 healthy volunteers and showed a significant negative correlation with chronological age (Supplementary Fig. 1), with spearman correlation coefficient *r* = − 0.7039**** (*p* < 0.001), this group was named as validation group. The TL showed significant change at the cut-offs of 40 and 70 years. Thus, the validation group was further divided into three sub-groups, with one sub-group consisting of healthy individuals of age between 1 to 39 years (young age), 40–69 (middle age) and ≥ 70 years (elderly). Supplementary Fig. 2 presents a box plot of three age groups showing a significant (***p < 0.001) decline in TL, in middle aged and elderlies as compare to younger groups. The longest TL or T/S ratio was measured at 2.46 and shortest was undetectable or 0 at the age range of 1 and 100 respectively, in healthy volunteers. Table 1 represents clears distinction between the TL and age in all three age groups. As expected, only two healthy volunteers out of 67 had shorter telomere of range between 0 and 0.62 T/S ratio, whereas majority of the volunteers had T/S ratio ranging from 1.5 to 2.46, 16 out of 19 middle aged individuals had T/S ratio of 0.62 to1.49 and since the number of volunteers was very low in age group of more than 70 year, so only 4 volunteers had T/S ratio ranging from 0 to 0.62 (Table 1).

### Correlation of telomere length with aging in healthy volunteers and metabolic syndrome patients

**Figure 1:**
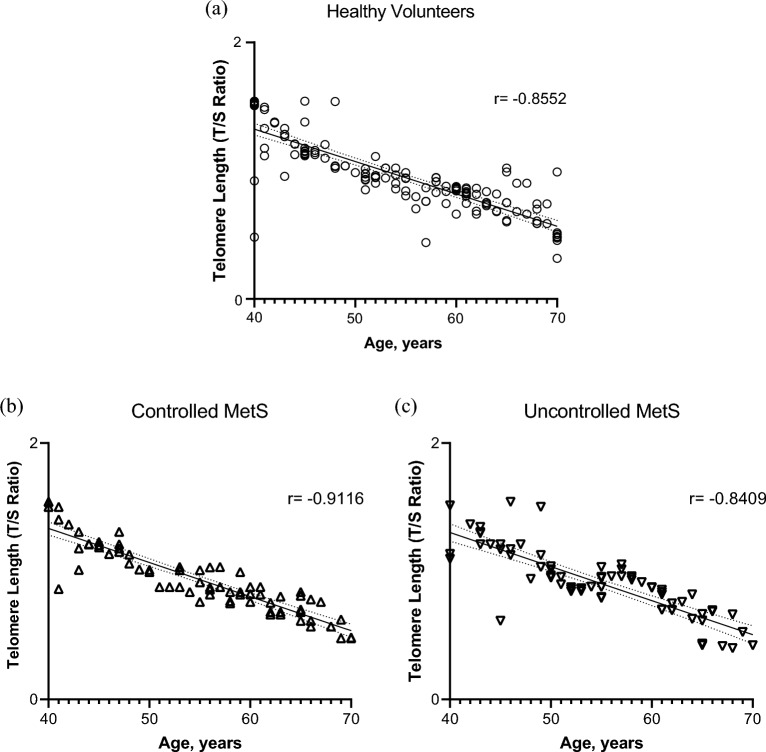
Correlation between telomere length (T/S ratio) and age in; (**a**), healthy volunteers (**b**), patients with controlled metabolic syndrome and (**c**) patients with un-controlled metabolic syndrome. A scatterplot showing a significant negative correlation between age (x-axis) and adjusted telomere length (y-axis), ****p < 0.001. The Spearman correlation coefficient (r) is indicated.

**Figure 2:**
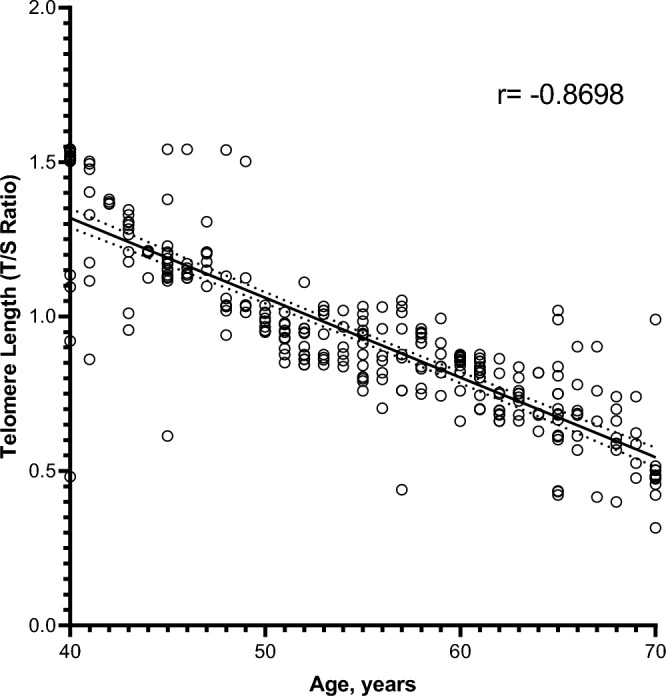
Correlation between telomere length (T/S ratio) in age matched healthy individuals and in patients with both controlled and un-controlled metabolic syndrome, at the age of 40–70 years. A scatterplot showing a significant negative correlation between selected age (x-axis) and adjusted relative telomere length (y-axis), ***p < 0.001. The Spearman correlation coefficient (r) is indicated.

A total of 298 patients recruited, including 149 age matched healthy volunteers, 72 with controlled metabolic syndrome and 77 uncontrolled syndrome. Significant negative correlation was found between TL and age, in all the three groups with the similar pattern of decline in TL (Fig. 1a–c). The analysis of the effect of MetS showed a significant negative correlation r = − 0.8698**, n = 298 (Fig. 2).

### Analysis of influence of metabolic syndrome on telomere length

**Figure 3:**
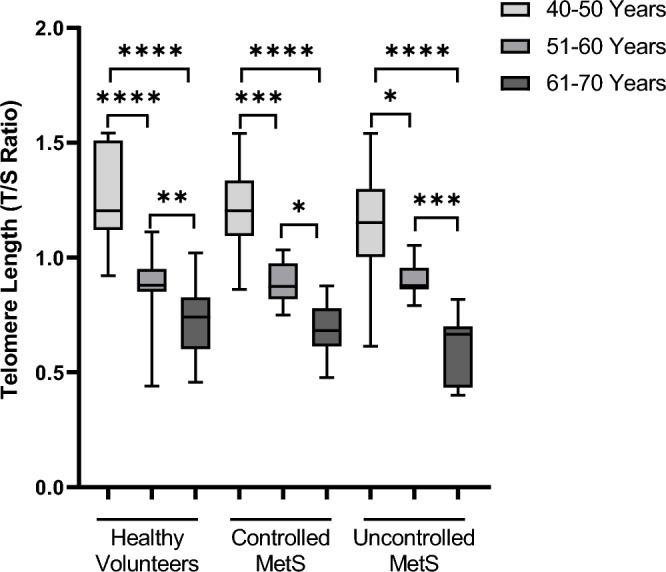
Comparison between telomere length (T/S ratio) in age matched healthy individuals and patients with both controlled and un-controlled metabolic syndrome, at the age segments ranging from 41-50y, 51–60 and 61–70 years. Kruskal-Willis test was found significant, ***p < 0.001, post hoc Dunn’s test was applied for multiple comparison as displayed in box plots showing significant differences, *p = 0.0332, **p = 0.0021, ****p = 0.0002, ****p < 0.0001.

To compare the combine effect of age and MetS on T/S ratio, TL total of 298 patients were divided into three groups, healthy volunteers, controlled and uncontrolled metabolic syndrome (Table 2). The T/S ratio was measured and was calculated on the basis of ten years age segments ranging from 41–50 years, 51–60 years and 61–70 years (Fig. 3). There was a significant difference in T/S ratio in age matched 61–70 years old patients with uncontrolled metabolic syndrome when compared to healthy volunteers. However there was no significant difference found in age groups 41–50 years and 51–60 years in all the three groups.

## Discussion

In this study, we determined TL levels for biological age determination, and identified three biological age groups including < 40 as younger population, middle age between 41 and 70 and elderly > 70 years with significant difference in the TL. The study also evaluated the impact of metabolic syndrome on TL showing a significant negative correlation with age, with limited impact of metabolic syndrome. Though uncontrolled metabolic syndrome causing relatively faster shortening.

Most of the studies performed in elderly, were related to shorten telomere, when compared to those from the younger population^16^. Age-related alteration in telomere length is a normal biological process^17,18^. Human telomere length appears to be shortening at a pace of 24.8 to 27.7 base pairs every year^17,18^. Individuals having telomere lengths that are shorter than the average age group are more likely to develop age-related diseases or also have a relatively shorter lifespan^19–21^. Previous studies have shown that age, genetic and epigenetic make-up, environment, social and economic status, exercise, body weight, and smoking are just a few of the factors that affect TL^17,22–25^. However, TL does not appear to be significantly influenced by sex^18^, once TL shorten enough, the cell goes into an irreversible change and does not divide anymore, hence become senescent and eventually undergoes apoptosis^26,27^. The results obtained in our study confirm shortening of TL in the elderly. This is novel study to determine cut-offs to define biological age based on TL, where the younger age group at 40, middle aged between 40 to 70 and elderly as ≥ 70 years of age. The TL may have strong association with physiological reserves which will help in prediction of survival in many chronic illnesses and may also help in determining the degree of aggressiveness in therapy for chronic illness such as cancer, heart failure and renal failure. Though there is limited literature available to comment till date. Given the complex physiology of aging and influence of multiple factors it is very likely that telomere length also get influenced by internal as well as external factors. Co-morbidities and environmental toxins play key role in expediting age. Therefore, it is also complex to determine a marker to assess biological age which is determinant of the physiological reserves, in turn can predict survival of the person so that management decision of chronic illnesses like cancer, cardiac and renal failure can be made wisely. Our study has however shown potential of telomere length to assess biological age and shown strong correlation with advancing age.

As discussed earlier, TL serves as a biological age indicator, for this Okuda et al*.*^28^ and Factor-Litvak et al.^29^ proposed TL from blood sample, which ranged from 8.5 to 13.5 kb and 7.0–11.6 kb. These TL shorten annually with the growing age, and at the age of 60 years the TL reaches about 5–6 kb^30^. However, in this study TL measured in healthy individuals which were divided into three different age groups, clearly defines the cut off values of TL and its negative correlation with advancing age. Replicative attrition leading to telomere shortening, though, it is not the only reason for age-dependent telomere shortening. Due to their chemical makeup, telomeres are extremely vulnerable to oxidative damage^31^. It is not unexpected that tendencies towards lower telomerase activity and increased telomere turnover have frequently been identified in individuals with MetS given that MetS is known to cause persistent oxidative stress and the inflammation^31^. However, the causal mechanisms of TL shortening and the disease pathogenesis behind TL shortening are not yet fully understood. Short telomeres have been linked to MetS in several studies, although results have been inconsistent.

Moreover, the interpretation of TL measurements is complicated by inter-individual differences influenced by various genetic, environmental, and lifestyle factors. For instance, differences in telomere attrition rates have been attributed to genetic variants affecting telomere maintenance mechanisms, such as telomerase activity or arginine methylation. In contrast, lifestyle factors, such as obesity, smoking, and physical activity, have also been linked to telomere length variation.

The study has strength of studying a considerable number of healthy population and provided cut-offs to identify age groups including younger (< 40), middle aged (41–70) and elderly (> 70) years).

However, healthy volunteers and cases were only evaluated on the basis of absence of metabolic syndrome and related disorders comprehensive health assessment was not done, where there is potential of some inflammatory conditions influencing telomere length. The patients were not followed-up for a longer period to look at the serial telomere lengths. Thus these are considered as limitations of the study.

In conclusion, TL remains a relevant and promising biomarker for health where it has shown potential to determine and differentiate younger and elderly population. Regardless of the presence or absence of MetS, the TL showed a significant negative correlation with aging, but interestingly untreated metabolic syndrome induces accelerated telomere shortening with the advance age as reflected in our findings.

Further validation studies are required to confirm its utility for clinical practice.

### Supplementary Information

Supplementary Figures.

## Data Availability

All data are available on request from the corresponding author (BMS).
