# Supplementary material for: Relationship between aging and control of metabolic syndrome with telomere shortening: a cross-sectional study
Source: Sci Rep. 2023 Oct 19;13:17878. doi: 10.1038/s41598-023-44715-1 (PMC10587132; doi:10.1038/s41598-023-44715-1)
Supplement: Supplementary file 1 — Supplementary Figures. [file 41598_2023_44715_MOESM1_ESM.pptx]

## Slide 1
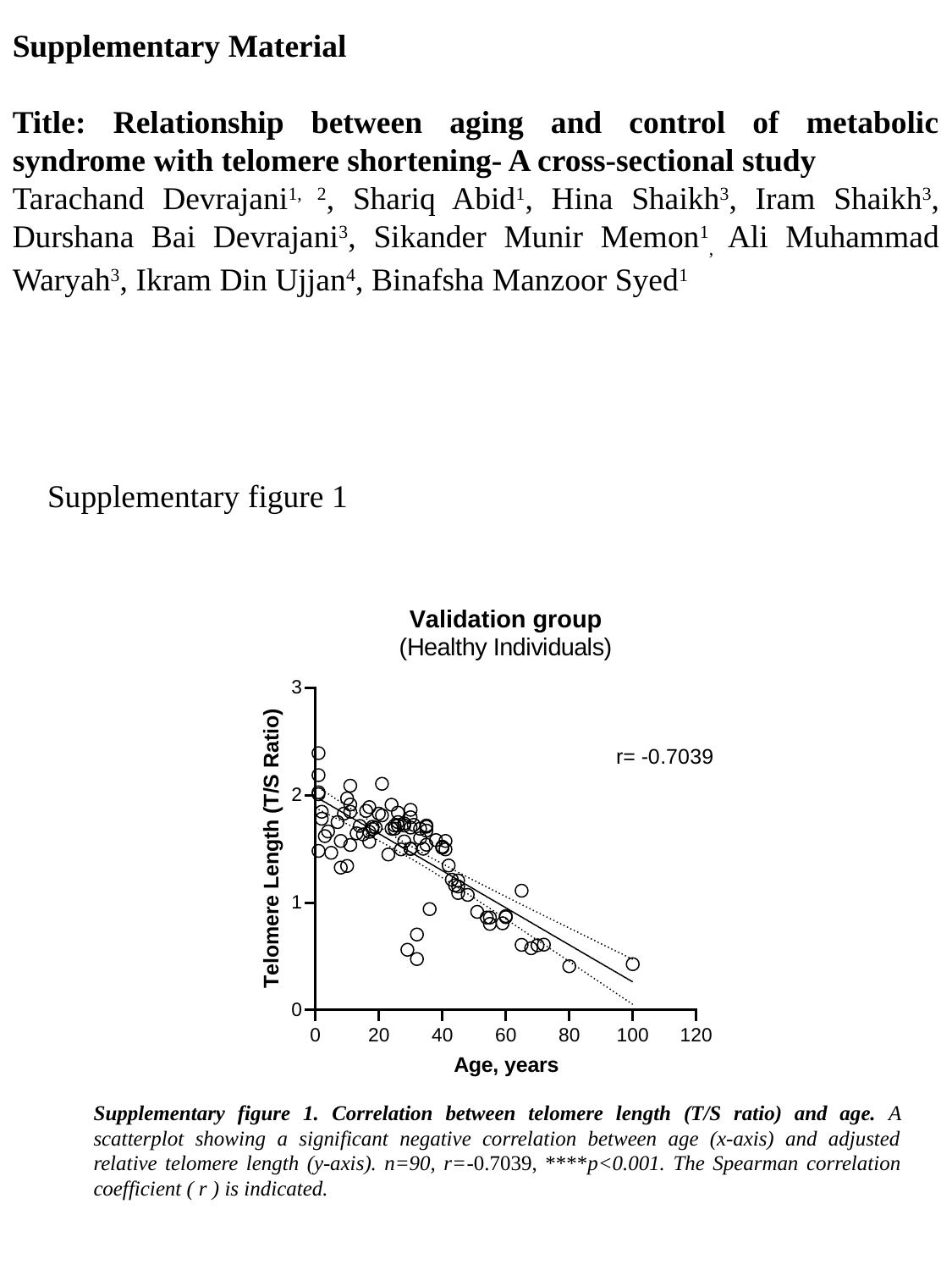

Supplementary Material
Title: Relationship between aging and control of metabolic syndrome with telomere shortening- A cross-sectional study
Tarachand Devrajani1, 2, Shariq Abid1, Hina Shaikh3, Iram Shaikh3, Durshana Bai Devrajani3, Sikander Munir Memon1, Ali Muhammad Waryah3, Ikram Din Ujjan4, Binafsha Manzoor Syed1
Supplementary figure 1
Supplementary figure 1. Correlation between telomere length (T/S ratio) and age. A scatterplot showing a significant negative correlation between age (x-axis) and adjusted relative telomere length (y-axis). n=90, r=-0.7039, ****p<0.001. The Spearman correlation coefficient ( r ) is indicated.

## Slide 2
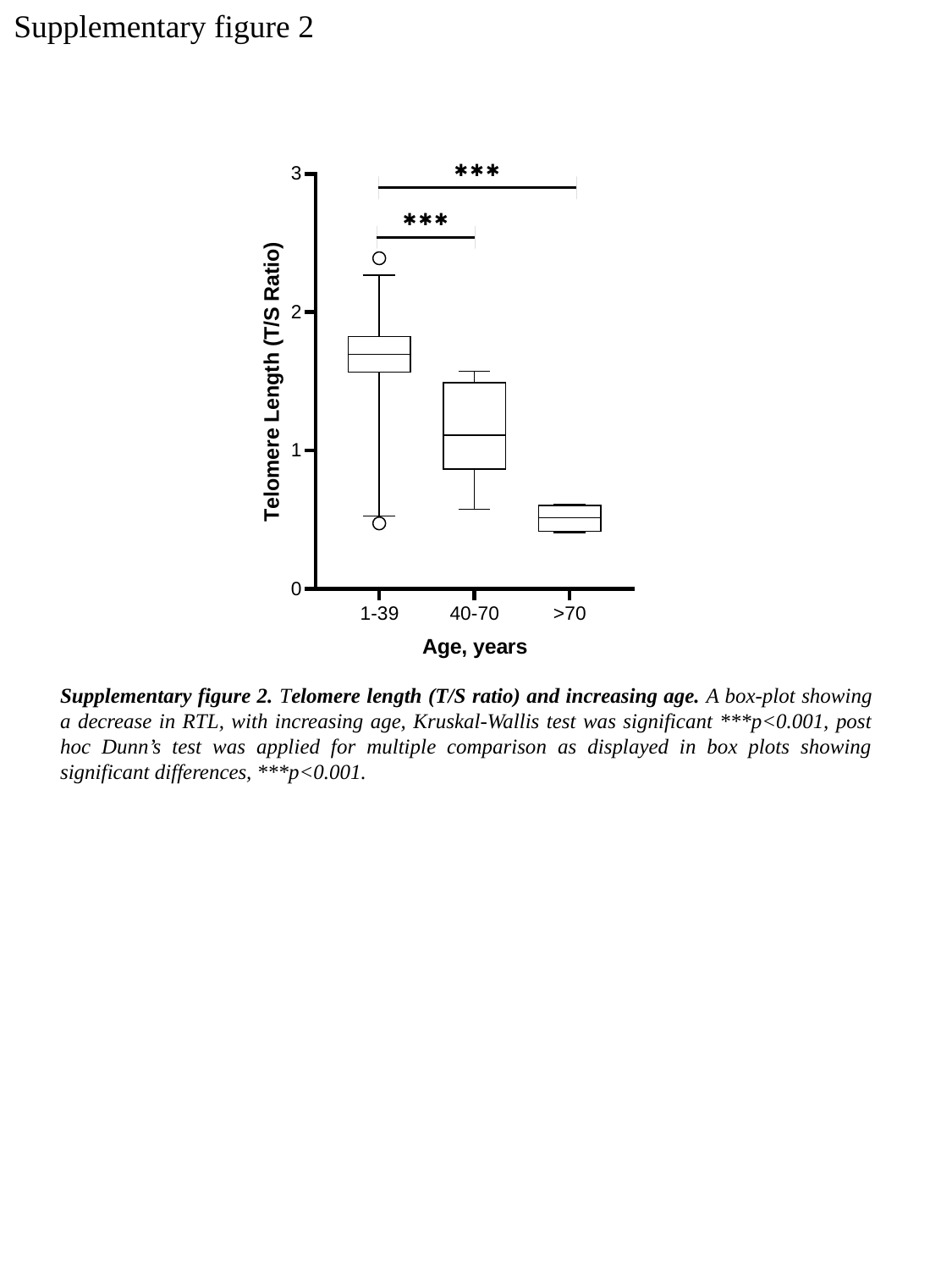

Supplementary figure 2
Supplementary figure 2. Telomere length (T/S ratio) and increasing age. A box-plot showing a decrease in RTL, with increasing age, Kruskal-Wallis test was significant ***p<0.001, post hoc Dunn’s test was applied for multiple comparison as displayed in box plots showing significant differences, ***p<0.001.
